# Sydenham Chorea With Elevated Interleukin-12 Levels Responsive to Plasmapheresis and Immunotherapy

**DOI:** 10.7759/cureus.100749

**Published:** 2026-01-04

**Authors:** Zhimin Xu, Jon Rosenberg, Robert Fekete, Anila M Thomas

**Affiliations:** 1 Neurology, New York Medical College, Valhalla, USA

**Keywords:** amantadine, plasmapheresis, rheumatic fever, streptococcal infection, sydenham chorea

## Abstract

Sydenham chorea (SC) presents with random abnormal involuntary movements that occur after an autoimmune reaction to a prior group A beta-hemolytic streptococcal infection. While most cases resolve spontaneously, some cases have a prolonged duration of symptoms and recurrences.

We discuss a 22-year-old woman who presented with a two-month history of involuntary, brief, random, and irregular movements of the limbs. She had a history of multiple streptococcal throat infections. At age two, she had scarlet fever. After ruling out other causes of chorea, she was diagnosed with Sydenham chorea. She was treated with intravenous immunoglobulin, oral prednisone, and amantadine, resulting in full symptom resolution. Interleukin-12 (IL-12) was elevated approximately seven months after hospital discharge.

Chronic elevation of IL-12 differs from previously published findings, which describe elevation only during the acute phase of the disease. Intensive immunosuppressive treatment during the acute phase, along with adherence to antibiotic therapy, may have contributed to the full resolution of her chorea.

## Introduction

Sydenham chorea (SC) presents with random abnormal involuntary movements that occur after an autoimmune reaction to a prior group A beta-hemolytic streptococcal infection [1]. SC is classically seen in children and adolescents and is one of the major clinical manifestations of acute rheumatic fever in the Jones criteria, making it an important marker of systemic, post-streptococcal autoimmunity. Clinically, patients typically present with generalized or asymmetric choreiform movements, hypotonia, and gait disturbance, often accompanied by behavioral or neuropsychiatric symptoms such as emotional lability, anxiety, or cognitive difficulties. The name of the disorder comes from the Greek word for “dance,” which is choreia (χορεία). Antibodies to the streptococcal infection mistakenly target the basal ganglia [2]. SC is associated with acute rheumatic fever, in which a similar mechanism leads to carditis, arthritis, subcutaneous nodules, and erythema marginatum. While most cases resolve spontaneously, there are cases with prolonged duration of symptoms and recurrences [3], necessitating further investigation into underlying immunological mechanisms. Severe or refractory cases, such as those in which chorea interferes with activities of daily living, may benefit from immunomodulatory therapies, such as corticosteroids, intravenous immunoglobulin (IVIG), or plasmapheresis. This case documents persistent elevation of interleukin-12 (IL-12) several months after symptom resolution, offering potential insights into the immunopathogenesis of SC.

This article was originally posted to the Research Square preprint server on July 15, 2025 [4].

## Case presentation

A 22-year-old woman presented to the Emergency Department (ED) on December 30, 2022, with a two-month history of involuntary, brief, random, and irregular movements of the limbs and slurred speech for one week. She had a history of scarlet fever at age two. Her symptoms included intermittent movements of the mouth and tongue, writhing movements of the right hand, and the right foot. The patient had a recent history of group A streptococcal throat infection diagnosed using a pharyngeal rapid antigen detection test and confirmed by culture in September 2022. She was treated with amoxicillin 500 mg orally twice daily for 10 days. In October, she presented to the ED with a fever (102 °F), neck pain, photophobia, and sore throat and was diagnosed with a viral respiratory infection. The following month, she began experiencing difficulty ambulating, difficulty performing tasks with her right hand, weight loss, fatigue, redness and warmth in her knuckles, and worsening speech difficulties. Her primary care physician ordered a brain MRI, which she was told was negative, and her broad constellation of symptoms was attributed to stress. She presented again to the ED due to worsening involuntary movements, which led to dropping objects she was holding.

Her past medical history was significant for multiple episodes of streptococcal pharyngitis, scarlet fever at age two, and attention-deficit/hyperactivity disorder (ADHD) treated with methylphenidate until age 14. Born in Egypt to American parents, she moved to the United States for college in 2018 and currently works as a teacher for autistic children, living with her grandparents and mother. Her family history was significant for Alzheimer’s disease in her maternal grandmother and mother, and a paternal half-sister with a history of Pediatric Autoimmune Neuropsychiatric Disorders Associated with Streptococcal Infections (PANDAS).

Physical examination revealed a systolic murmur at the apex. Oropharyngeal examination revealed no exudates. The tonsils were not swollen, and the neck was supple. Psychiatric assessment indicated increased anxiety and irritability. She expressed a paranoid belief that nurses would discharge her in the middle of the night. Neurological examination was significant for mildly slurred speech, mouth dyskinesias, tongue darting, and right-hand writhing movements that changed throughout the encounter. Video 1 demonstrates these movements. A milkmaid sign was present when attempting a hand grip. She also exhibited hand spooning (wrist arching and hyperextension of the fingers), elbow flexion when asked to lift her arms (touchdown sign), and an abnormal gait with intermittent sideways movement of the right foot. Her hyperkinetic movements were consistent with chorea. 

**Video 1:** Chorea and motor impersistence Bilateral upper extremity, head, face, and tongue chorea with motor impersistence. Video published with consent from the patient.

Throat culture obtained in the ED revealed beta-hemolytic streptococcus, non-group A. Prior throat culture results performed at different hospitals were not available. Blood cultures were negative. Serum anti-streptolysin O testing was negative.

Lumbar puncture revealed cerebrospinal fluid (CSF) protein of 20 mg/dL, lactic acid of 1.0 mmol/L, glucose of 58 mg/dL, and no oligoclonal bands. The CSF red blood cell count was 1/mm³. The CSF white blood cell count was 113/mm³, with 82% lymphocytes, 12% monocytes, and 6% polymorphonuclear cells. CSF bacterial culture was negative (Table 1). 

**Table 1:** Results of CSF and serum studies Results of CSF and serum studies with normal ranges. CSF: cerebrospinal fluid, WBC: white blood cell, RBC: red blood cell, PML: polymorphonuclear leukocytes, NEG: negative, DNASE B: deoxyribonuclease B, IgG: immunoglobulin G, IgM: immunoglobulin M, TSH: thyroid-stimulating hormone, CRP: C-reactive protein, hs-CRP: high-sensitivity C-reactive protein.

Laboratory Studies	Results	Normal Value Range and Units
CSF biochemistry		
Glucose	58	50-80 mg/dL
Protein	20	15-45 mg/dL
Lactic acid	1.0	<2.1 mmol/L
CSF cell count with differentials		
WBC	113	0-5/mm^3^
RBC	1	0/mm^3^ (tube sample #2)
PML	6	0-7%
Lymphocytes	82	28-96%
Monocytes	12	16-56%
CSF cultures and microbiology		
Gram stain	NEG	NEG
Bacterial culture	NEG	NEG
Serum studies		
Blood culture	NEG	NEG
Anti-streptolysin O	NEG	NEG
DNASE B antibody	272	<301 units/mL
Anti-cardiolipin IgG	1.88	<15 units/mL
Anti-cardiolipin IgM	6.51	<12.5 units/mL
Anti-nuclear antibody	1:80 (speckled)	<1:40
Anti-double-stranded DNA antibody	<12.3 units	<30 units
Thyroid-stimulating hormone	2.183	0.350-4.700 mIU/L
C-reactive protein	<0.10	<0.10 mg/dL
24-hour urine copper	<40 µg/12h	<40 µg/12h
Ceruloplasmin	15	<51 mg/dL
High sensitivity C-reactive protein at hospital discharge	3.6	<3.1 mg/L
Interleukin-12 at follow-up	3.9 pg/mL	≤1.9 pg/mL

Anti-cardiolipin antibody IgG was negative at 1.88 units/mL (normal <15 units/mL). Anti-cardiolipin antibody IgM was negative at 6.51 units/mL (normal <12.5 units/mL). Antinuclear antibody was positive at a titer of 1:80. Anti-double-stranded DNA antibody was negative at <12.3 units (normal <30 units). Serum thyroid-stimulating hormone was normal at 2.183 mIU/L (normal 0.350-4.700 mIU/L).

The Mayo Clinic Laboratories (Rochester, MN, USA) paraneoplastic antibody panel was negative in both cerebrospinal fluid (CSF) and serum for amphiphysin, AGNA-1, ANNA-1, ANNA-2, ANNA-3, CASPR-2, CRMP-5, DPPX, GAD65, GRAF1, IgLON5, ITPR1, KLHL11, LGI1, mGluR1, NIF, NMDA-R, PCA-1, PCA-2, and PCA-Tr antibodies. The serum was also negative for P/Q-type calcium channel antibody (Table 2). 

**Table 2:** Paraneoplastic studies in CSF and serum CSF: cerebrospinal fluid, Ab: antibody, IFA: indirect immunofluorescence assay, NEG: negative, AGNA-1: anti-glial nuclear antibody type 1, ANNA: anti-neuronal nuclear antibody, CASPR2: contactin-associated protein-like 2, CBA: cell-binding assay, CRMP5: collapsin response mediator protein 5, DPPX: dipeptidyl-peptidase-like protein 6, GAD-65: glutamic acid decarboxylase 65, RIA: radioimmunoassay, GRAF1: GTPase regulator associated with focal adhesion kinase 1, ITPR1: inositol 1,4,5-trisphosphate receptor type 1, KLH11: kelch-like protein 11, LGI1: leucine-rich glioma-inactivated 1, mGluR1: metabotropic glutamate receptor 1, NIF: neuronal intermediate filament, NMDA-R: N-methyl-D-aspartate receptor, IgG: immunoglobulin G, P/Q-type: P/Q-type voltage-gated calcium channel.

Variables	Methodology	Results	Normal Range Values and Units
CSF paraneoplastic antibody			
Amphiphysin Ab	IFA	NEG	NEG
Anti-glial nuclear Ab, Type I (AGNA-1)	IFA	NEG	NEG
Anti-neuronal nuclear Ab (ANNA), Type I-II-III	IFA	NEG	NEG
CASPR2-IgG	CBA	NEG	NEG
CRMP5-IgG	IFA	NEG	NEG
DPPX Ab	IFA	NEG	NEG
GAD-65 Ab assay	RIA	0	≤0.02 nmol/L
GRAF1 Ab	IFA	NEG	NEG
IgLON5 Ab	CBA	NEG	NEG
ITPR1 Ab	IFA	NEG	NEG
KLH11 Ab	CBA	NEG	NEG
LGI1-IgG	CBA	NEG	NEG
mGluR1 Ab	IFA	NEG	NEG
NIF	IFA	NEG	NEG
NMDA-R Ab	CBA	NEG	NEG
Purkinje cell cytoplasmic Ab Type I, II, Tr	IFA	NEG	NEG
Serum paraneoplastic antibody			
AGNA-1	IFA	NEG	NEG
Amphiphysin Ab	IFA	NEG	NEG
Anti-neuronal nuclear Ab (ANNA), Type I-II-III	IFA	NEG	NEG
CASPR2-IgG	CBA	NEG	NEG
CRMP5-IgG	IFA	NEG	NEG
DPPX Ab	CBA	NEG	NEG
GAD-65 Ab assay	RIA	0	≤0.02 nmol/L
GRAF1 Ab	IFA	NEG	NEG
IgLON5	CBA	NEG	NEG
ITPR1	IFA	NEG	NEG
KLH11 Ab	CBA	NEG	NEG
LG1-IgG	CBA	NEG	NEG
mGluR1 Ab	IFA	NEG	NEG
NIF Ab	IFA	NEG	NEG
NMDA-R Ab	CBA	NEG	NEG
P/Q-type calcium channel	IFA	NEG	NEG
Purkinje cell cytoplasmic Ab Type I, II, Tr	IFA	NEG	NEG

**Figure 1:**
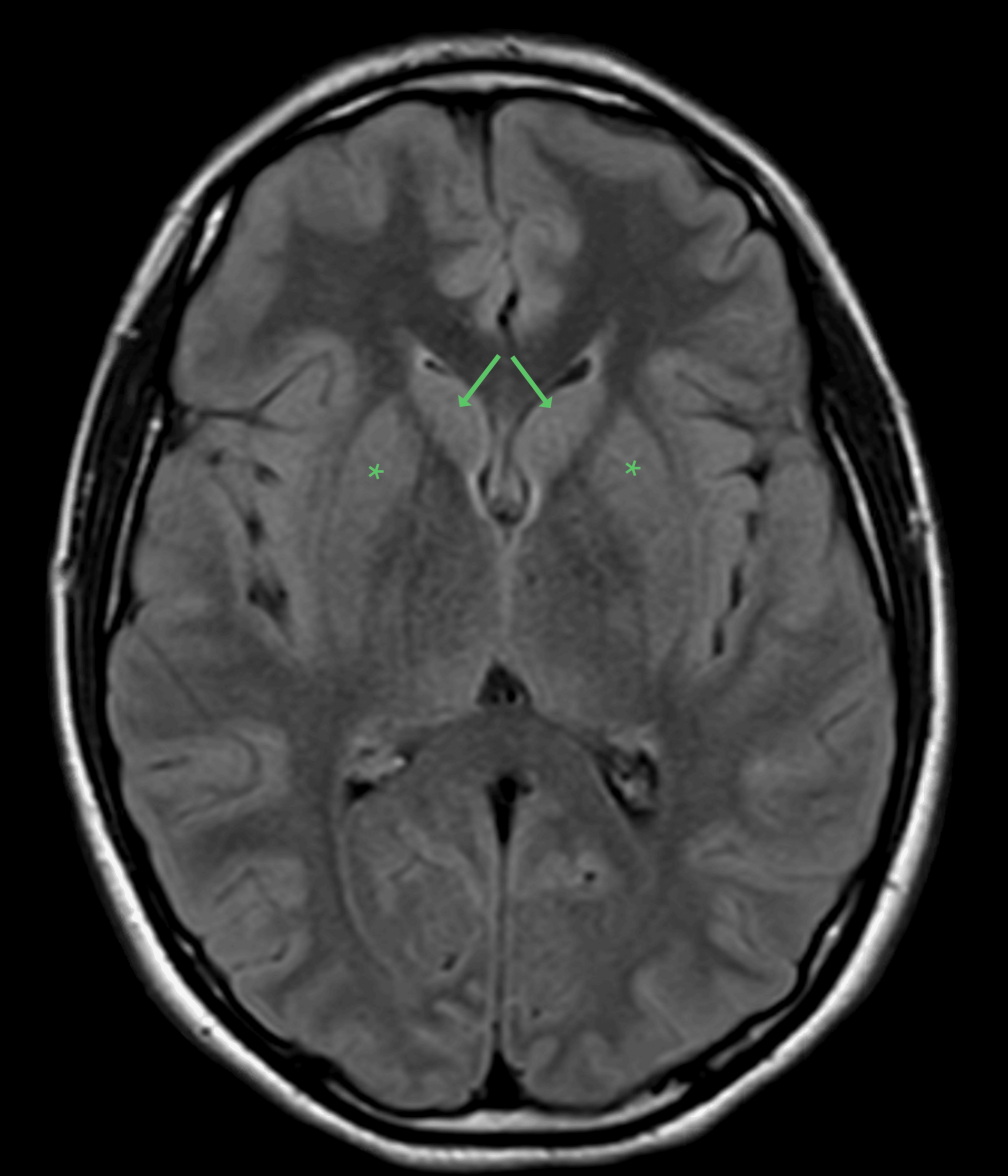
Axial MRI MRI Fluid Attenuation Inversion Recovery (FLAIR) image demonstrating enlarged caudate nuclei bilaterally (arrows) and mildly hyperintense bilateral putamina (stars) due to Sydenham's chorea.

**Figure 2:**
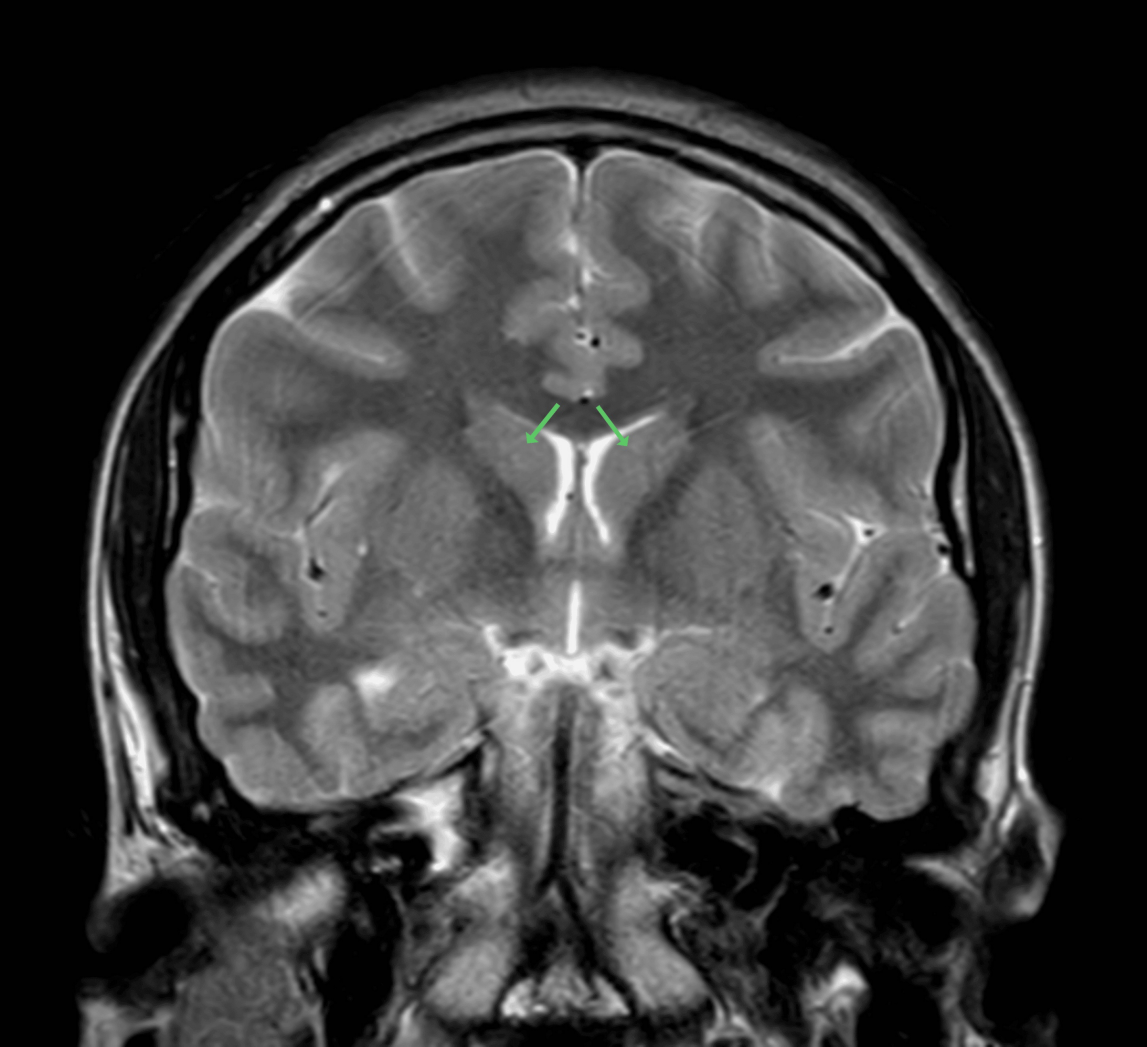
Coronal MRI MRI T2 image indicating enlarged caudate nuclei bilaterally (arrows) as a result of Sydenham's chorea.

C-reactive protein in the ED was low at <0.10 mg/dL. Serum ceruloplasmin was low at 15 mg/dL. Twenty-four-hour urine copper was negative. Brain MRI performed during hospitalization revealed normal to mildly hyperintense caudate nuclei, which appeared mildly swollen (Figures 1, 2). She received five plasmapheresis treatments during hospitalization, after which she was discharged. Serum high-sensitivity C-reactive protein (hsCRP) at discharge was elevated to 3.6 mg/L. Her chorea was treated with amantadine 100 mg orally daily for three weeks. Prednisone 60 mg daily (1 mg/kg) was initiated on day five of hospitalization for one week, followed by 50 mg daily for two weeks. The dosage was subsequently reduced by 10 mg every week.

**Figure 3:**
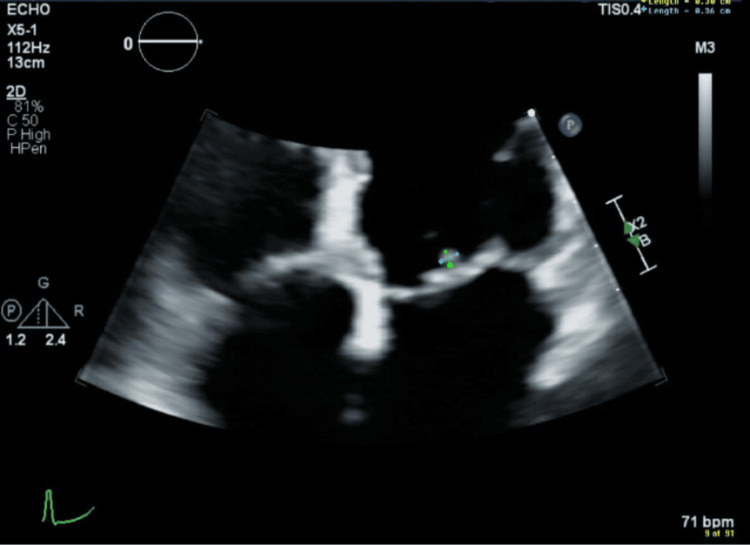
Echocardiogram Echocardiogram image showing questionable early vegetation (outlined in green) versus mild thickening of mitral valve leaflets.

The patient presented for outpatient follow-up in July 2023. Her chorea had completely resolved. She was compliant with a chronic oral penicillin VK prescription of 250 mg twice daily. Interleukin-12 (IL-12) at this time was elevated to 3.9 pg/mL (normal ≤1.9 pg/mL). Her transthoracic echocardiogram showed moderate eccentric mitral regurgitation and a small vegetation versus mild thickening of both mitral leaflets at the tips, possibly involving the subvalvular apparatus (Figure 3). The patient did not wish to undergo transesophageal echocardiography for further evaluation of possible small valvular vegetation.

## Discussion

Diagnosing SC is mostly clinical, with the presence of characteristic neurological and behavioral symptoms following streptococcal infection. Supporting evidence includes elevated antistreptolysin O titers and other streptococcal antibodies, although their absence does not preclude the diagnosis [5]. Neuroimaging is typically normal but is valuable for excluding other conditions. The Jones criteria are relevant for a comprehensive diagnosis in the presence of other acute rheumatic fever manifestations.

The differential diagnosis includes hereditary chorea, structural or vascular brain abnormalities, other autoimmune disorders, infections, metabolic disorders, and toxin exposure. Particularly notable is PANDAS, which shares the autoimmune response to streptococcal infection with SC but typically presents with abrupt-onset OCD or tic disorders. Careful distinction between SC and PANDAS is crucial for appropriate management [6].

The treatment of SC focuses on symptom management and recurrence prevention. Dopamine receptor antagonists (e.g., haloperidol) and antiseizure medications (e.g., valproic acid) are commonly used for chorea. In this case, amantadine was helpful. Severe or refractory cases may benefit from immunomodulatory therapies, such as corticosteroids, intravenous immunoglobulin (IVIG), or plasmapheresis. Our patient received corticosteroids, as well as plasmapheresis, due to significant chorea that led to difficulty grasping objects normally. Long-term antibiotic prophylaxis, primarily with penicillin, is important for preventing recurrent streptococcal infections and subsequent clinical episodes [7].

Twenty-four-hour urine copper was low, which argues against Wilson’s disease. MRI of the brain did not show any correlates of Wilson’s disease, such as the panda sign. High-sensitivity CRP (hsCRP) at discharge was elevated to 3.6 mg/L and returned to normal at 0.4 mg/L about 2.5 months after discharge, which is consistent with resolution of symptoms.

In this patient, IL-12 was elevated to 3.9 mg/dL at seven months postdischarge, despite clinical resolution of SC. Although acute-phase IL-12 levels were not obtained after the initial ED presentation, which is a limitation of this case report, the delayed elevation contrasts with prior reports showing IL-12 elevation only during the acute phase but not in persistent SC [8] using ELISA methodology from Amersham Pharmacia UK. Our IL-12 serum test was performed by ARUP Laboratories using magnetic bead technology, which has a different lower limit of detection (LLOD) and normal value, limiting comparability [9,10].

IL-12 has a complex proinflammatory as well as immunoregulatory function [11]. Interleukin 12 was originally called Natural Killer Cell Stimulatory Factor (NKSF) [12]. It promotes Th1 differentiation, increases activity of NK and T cells, and stimulates production of interferon-gamma [13]. Increased IL-12 and Th1 activity have been implicated in the pathology of multiple sclerosis [14].

Increased interferon-gamma signaling induces the secretion of CXCL9, a chemokine that attracts immune cells. CXCL9 is also called MIG (monokine induced by interferon-gamma), given its production by monocytes and macrophages. Recruitment of T cells into the brain via CXCL9 has been shown in murine experiments [15]. In ischemic stroke, CXCL9 works to break down the blood-brain barrier, which leads to edema [16]. Increased sizes of the caudate, putamen, and globus pallidus have been reported in Sydenham’s chorea on MRI imaging [17]. Hence, there is a theoretical basis for IL-12 activity to be involved in symptomatic neurological autoimmune disease via increased Th1 activity, chemoattraction of T cells, blood-brain barrier breakdown, and edema.

## Conclusions

The key findings of this case include persistent elevation of IL-12 to 3.9 pg/mL (reference ≤1.9 pg/mL) seven months after presentation, swelling of the caudate nuclei and putamina on brain MRI during the acute phase, and complete resolution of chorea with aggressive immunotherapy.

The patient received five sessions of plasmapheresis, amantadine 100 mg orally once daily for three weeks, and oral prednisone starting at 60 mg/day, tapered weekly. Her symptoms resolved within three months of initial presentation. Brain MRI revealed mildly hyperintense and swollen caudate nuclei without broader basal ganglia involvement. These findings support the hypothesis that IL-12 may contribute to a Th1-dominant immune response and T-cell chemotaxis, potentially leading to blood-brain barrier disruption and striatal edema, as suggested in other neuroinflammatory models.

Limitations of the report include the absence of an acute phase IL-12 level, different LLOD and normal IL-12 values compared to other methodologies, and that data from one patient are not generalizable. Further prospective studies should consider serial cytokine profiling at presentation and follow-up using standardized assays to clarify the immunopathogenesis of both acute and persistent SC.
